# The impact of pain from medial meniscus injuries on walking movement patterns

**DOI:** 10.3389/fbioe.2025.1545521

**Published:** 2025-04-22

**Authors:** Yiyan Chen, Liyan Wang, Zhiying Fan, Haibin Zhou, Aming Lu

**Affiliations:** ^1^ Department of Physical Education, Suzhou Vocational University, Suzhou, China; ^2^ Department of Orthopedics, The Second Affiliated Hospital of Soochow University, Suzhou, China; ^3^ Physical Education and Sports School, Soochow University, Suzhou, China

**Keywords:** medial meniscus injury, pain, movement patterns, biomechanics, inertial measurement units

## Abstract

**Background:**

Existing literature provides inconclusive evidence regarding the impact of pain on movement patterns, especially in medial meniscus injuries. This study investigated how pain induced by medial meniscus injuries affects walking movement patterns, focusing on the biomechanical mechanisms. The goal was to develop targeted rehabilitation.

**Methods:**

Thirty control participants (15 male, 15 female), 23 individuals with medial meniscus injury but no pain (11 male, 12 female), and 51 individuals with medial meniscus injury and pain (24 male, 27 female) were recruited. Gait data was collected using eight inertial measurement units and a video camera. Pain characteristics were assessed using the Visual Analog Scale (VAS) score, Tampa Scale for Kinesiophobia (TSK), Pain Catastrophizing Scale, and pain duration score. Statistical analyses were conducted using a one-way ANOVA to compare movement patterns among the three groups. Bivariate correlation analyses were performed within the pain group to examine the relationship between pain characteristics and movement patterns. The *p* was set at 0.05.

**Results:**

(1) ANOVA among the groups revealed significant differences (*p* < 0.05) in several parameters: a shorter swing phase, reduced hip and knee angles, increased variability index, increased calf-foot mean absolute relative phase (MARP) during the support phase, and decreased calf-foot MARP during the swing phase were associated with pain. (2) Within the pain group, pain in knee extension (PKE) was negatively correlated with hip and ankle angles, stride length, and thigh-calf MARP during the support phase (*p* < 0.05). The VAS exhibited a negative correlation with knee angle and stride length, and a positive correlation with shock attenuation (*p* < 0.05). The TSK showed a positive correlation with hip and knee angles, and calf-foot MARP during the swing phase, while it was negatively correlated with stride length (*p* < 0.05).

**Conclusion:**

Medial meniscus injury-induced pain has several adverse effects, including prolonged walking swing periods, reduced angulation, and increased variability while positively influencing coordination and shock attenuation. Pain intensity, kinesiophobia, and pain freedom contribute to these changes. Therapists should focus on pain management and movement pattern retraining to develop personalized rehabilitation. The angle of the swing phase should be emphasized during retraining.

**Clinical Trial Registration:**

https://www.chictr.org.cn/showproj.html?proj=65961, identifier ChiCTR2000041087

## 1 Introduction

Menisci, crescent-shaped fibrocartilaginous structures within the knee joint, comprise the medial and lateral menisci. These essential components of the knee joint serve crucial functions, including shock absorption, joint surface congruency, and joint stabilization. However, due to their structure and anatomical location, menisci are prone to injury. The literature suggests an incidence of approximately 60–70 meniscal injuries per 10,000 individuals (Majewski et al., 2006). The medial meniscus, with its greater number of attachments in both the root and body, exhibits a higher susceptibility to injury than the lateral meniscus (Markes et al., 2020). Meniscal injuries often present with symptoms such as knee pain, swelling, and stiffness. Among these, pain significantly impacts daily life. Pain, as defined by the International Association for the Study of Pain, is “An unpleasant sensory and emotional experience associated with actual or potential tissue damage, or described in terms of such damage” (Raja et al., 2020). It can significantly hinder daily activities, exercise, and mental health (Fukushima et al., 2023; Luque-Suarez et al., 2019; Kapos et al., 2024).

Movement patterns, characterized by temporal, spatial, and spatio-temporal attributes of a motor object’s movements during a task, can be considered neural traces. They are categorized into basic functional and sports-specific patterns. Walking is the most common mode of basic functional movement, according to the characteristics of human life (Liu, 2016). Pain alters motor control through neuromuscular changes, which can lead to modifications in movement patterns (Kantak et al., 2022). Knee pain intensity has been linked to movement pattern modifications, with previous studies showing correlations between knee pain intensity and center of pressure excursions and dynamic knee valgus in knee injury patients (Yalfani et al., 2024a; Yalfani et al., 2024b). Experimental knee pain has also been shown to impair joint moments and force development rates during isometric and isotonic muscle activations (Rice et al., 2019; Gallina et al., 2024). While research on lateral meniscus injuries has revealed pain-induced alterations in obstacle navigation (Chen et al., 2022), studies on the more common medial meniscus injuries remain limited.

Indeed, the experience of pain is multifaceted, encompassing more than just pain intensity. Key metrics in clinical research and social psychology, such as pain freedom, pain psychology, and pain duration, offer a more comprehensive understanding of pain and can mitigate the limitations of relying solely on pain intensity. However, the specific influence of these factors on movement patterns, particularly in the context of medial meniscus injuries, remains unclear. While some studies suggest that pain-related psychological states and pain duration can impact movement patterns (Suzuki et al., 2020; Norheim et al., 2019; Ohji et al., 2023; Souza de Vasconcelos et al., 2023; Nedder et al., 2024), others have found no significant effect (Priore et al., 2019; Amhamed et al., 2016). Despite the prevalence of pain-related fear of movement among patients with knee injuries (Machado et al., 2022; Bullock et al., 2022; Eymir et al., 2022), previous research has not directly addressed medial meniscus injuries. Patients with medial meniscus injuries demonstrate reduced walking speed, diminished lower extremity joint angles, and decreased variability in angular parameters during ambulation (Li et al., 2019; Magyar et al., 2012). Therefore, further investigation is necessary to determine whether pain characteristics, including pain intensity, psychological factors, and duration, directly contribute to altered movement patterns in this specific population. In addition to the limited literature on meniscus injuries, existing studies have not systematically collected data on the pain characteristics of a specific patient population and established connections between these characteristics and movement patterns. This gap may lead to incomplete treatment strategies and hinder therapists from implementing targeted interventions. This paper correlates the pain characteristics of patients with medial meniscus injuries to their movement patterns, providing a solid theoretical foundation for the development of rehabilitation programs. Therapists can utilize these findings to facilitate pain relief or selectively retrain movement patterns based on the results.

Traditional biomechanical evaluation instruments mainly rely on three-dimensional optical motion capture systems, such as Vicon (Germany) and Motion (U.S.), which have more stringent requirements for the use of the scene and higher costs. They also require systematic training of professionals in the operation of the system and are mostly used in professional laboratories. Compared with traditional biomechanical assessment instruments, wearable inertial measurement units have the advantages of being portable, non-invasive, low-cost, and not subject to site constraints. They can be used as biofeedback devices in movement pattern retraining in the later stage, which can reduce the cost of rehabilitation, improve the practicality of biofeedback, and provide a new way of thinking for remote and at-home rehabilitation. Based on this, IMUs were chosen as the assessment instrument in this study, aiming to enrich the practical prospects of IMUs in biomechanics and rehabilitation.

This study aimed to investigate the impact of knee pain on movement patterns during walking in patients with medial meniscus injuries, focusing on the biomechanical mechanisms of movement. We hypothesized that the presence of pain would influence knee angle and coordination during walking in these patients, and that pain-related fear would contribute to the manifestation of these changes. By investigating the impact of pain on walking movement patterns, we sought to enhance the understanding of pain-induced alterations in movement and provide a reference for the retraining of movement patterns. This study also aims to provide insights for rehabilitation therapy in patients with medial meniscus injuries.

## 2 Methods

### 2.1 Participants

From December 2020 to December 2021, 74 patients (35 males and 39 females) with unilateral medial meniscus injuries were recruited from the Department of Joint Surgery at the Second Affiliated Hospital of Soochow University. All participants were active hospital attendees who volunteered to take part in the study. Participants met specific inclusion and exclusion criteria.

Inclusion criteria were: (1) MRI confirmation of a medial meniscus injury in all patients; (2) absence of knee pathology other than the medial meniscus injury; (3) no history of previous knee surgery; (4) age between 18 and 60 years; (5) body mass index (BMI) less than 30 kg/m^2^; and (6) the ability to walk independently. Exclusion criteria were: (1) musculoskeletal injuries affecting areas of the body other than the knee; (2) central or peripheral neurological disorders; (3) unstable angina; (4) myopia exceeding 500 diopters without vision correction (Magyar et al., 2012); (5) use of pain medication within the 7 days preceding the test; (6) participation in rehabilitation within the past 6 months (Priore et al., 2019); and (7) elite athletes.

In this study, a visual analog scale (VAS) of 30 mm was established as a threshold to differentiate between patients experiencing definite pain and those who were not. This criterion for defining pain has been established as robust and scientifically valid (Norheim et al., 2019). Among the 74 participants, 51 individuals (24 males and 27 females) reported a knee VAS exceeding 30 mm in their daily lives over the past 7 days, meeting the criteria for inclusion in the pain group (Norheim et al., 2019; Priore et al., 2019). In contrast, 23 participants (11 males and 12 females) with a VAS of 0 mm were classified as the pain-free group. The source of pain was identified as a medial meniscus injury through physical examinations, including McGrath’s sign, conducted by a physician.

A matched group of 30 healthy individuals, comprising 15 males and 15 females, served as the control group. There were a total of 104 participants in this study, and basic information about each subgroup is presented in Table 1. Statistical analyses revealed no significant differences between the three groups in terms of gender, age, height, weight, and BMI, except for the VAS. Furthermore, analyses of disease duration and affected limb side in both the pain-free and pain groups yielded no significant differences.

**TABLE 1:** Basic information sheet for participants (x̄ ± s).

Indicators (n = 104)		Control group (n = 30)	Pain-free group (n = 23)	Pain group (n = 51)	*p*
Male/Female		15/15	11/12	24/27	0.344
Age (yr)		35.37 ± 9.41	35.35 ± 8.85	40.27 ± 10.76	0.057
Height (m)		1.66 ± 0.10	1.65 ± 0.08	1.64 ± 0.09	0.717
Weight (kg)		64.43 ± 8.34	63.83 ± 8.32	65.50 ± 9.92	0.714
BMI (kg/m^2^)		23.34 ± 2.22	23.45 ± 2.58	24.17 ± 2.78	0.317
VAS score (mm)		0.00 ± 0.00	0.00 ± 0.00	55.69 ± 15.38	0.000*
Course of disease (months)		0.00 ± 0.00	6.14 ± 5.31	6.77 ± 5.93	0.713
Affected limb	Left	—	9 (39.13%)	21 (41.18%)	0.869
	Right	—	14 (60.87%)	30 (58.82%)	

* indicates statistical significance.

Participants and their families provided informed consent for the pilot test and signed an informed consent form. Participants were informed of the potential hazards associated with the test, including the risk of inadvertent falls while walking. Additionally, they were made aware that no financial compensation would be provided. The study protocol was reviewed and approved by the Ethics Committee of the Second Affiliated Hospital of Soochow University (No. JD-LK-2021–017-01).

### 2.2 Instruments

**FIGURE 1:**
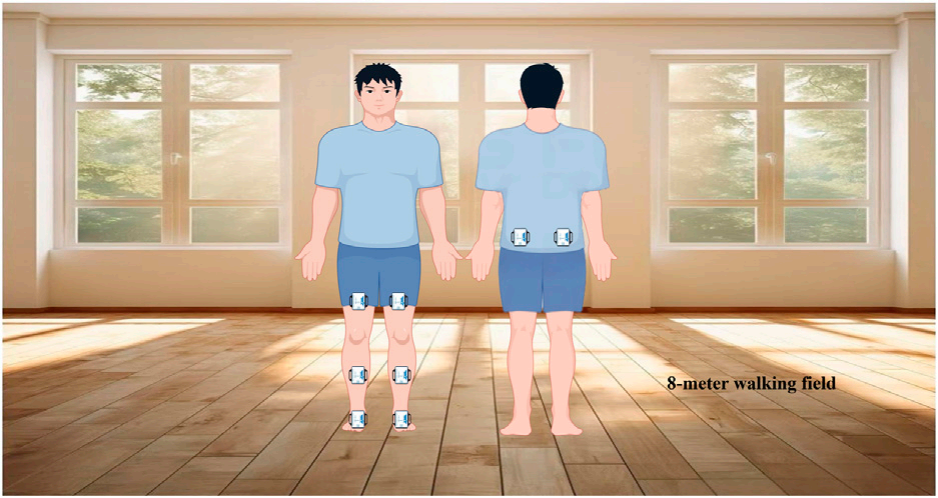
Schematic diagram of data acquisition process (By Figdraw).

In this study, IMUs were utilized to collect participant data. IMUs are small, lightweight, portable, box-shaped devices typically worn with a wide elastic band over the body’s motor segments. They connect to a computer and transmit data in real time to the relevant software. The advantages of IMUs include low cost, non-invasiveness, low energy consumption, ease of wear, minimal requirements for the testing environment, and straightforward operation. Eight IMUs (51.3 mm × 36 mm × 15 mm, model WT901BLEC, Vitor Technology Co., China) were utilized in this test (Wang et al., 2020). They were positioned on the waist, anterior thigh, anterior calf, and instep of the participants (see Figure 1; Table 2) (Chen et al., 2022). The IMUs were connected to MiniIMU software (China) for data reception, with a sampling frequency set to 50 Hz. A normal-speed video camera (model HDR-XR500E, released in 2009 by Sony, Japan) recorded the participants’ lower limb movements from the side, also at a sampling frequency of 50 Hz. SIMI Motion software (v9.2.2, Germany) was used to analyze the videos for motion tracking.

**TABLE 2:** Locations of the IMUs.

No.	Placement	Description
1	Left waist	Transverse to the 3rd lumbar vertebra, the XYZ axis of IMU corresponds to the sagittal plane, horizontal plane and coronal plane respectively
2	Anterior surface of left thigh	At 1/2 of the thigh, the XYZ axis of IMU corresponds to the sagittal plane, horizontal plane and coronal plane respectively
3	Anterior surface of left calf	At 1/2 of the calf, the XYZ axis of IMU corresponds to the sagittal plane, horizontal plane and coronal plane respectively
4	Left instep	At 1/2 of the instep, the XYZ axis of IMU corresponds to the sagittal plane, coronal plane and horizontal plane respectively
5	Right waist	Transverse to the 3rd lumbar vertebra, the XYZ axis of IMU corresponds to the sagittal plane, horizontal plane and coronal plane respectively
6	Anterior surface of right thigh	At 1/2 of the thigh, the XYZ axis of IMU corresponds to the sagittal plane, horizontal plane and coronal plane respectively
7	Anterior surface of right calf	At 1/2 of the calf, the XYZ axis of IMU corresponds to the sagittal plane, horizontal plane and coronal plane respectively
8	Right instep	At 1/2 of the instep, the XYZ axis of IMU corresponds to the sagittal plane, coronal plane and horizontal plane respectively

### 2.3 Experimental program

#### 2.3.1 Assessment of muscle strength and dynamic postural control

All patients were assessed for lower limb muscle strength and dynamic postural control to ensure baseline uniformity. Manual Muscle Testing (MMT) was conducted on the participants, with the British Medical Research Council Scale (BMRCS) showing a strength score of 5 for the quadriceps and hamstring on the healthy side, compared to a score of 4 on the affected side. The lateral single-leg squat, a straightforward clinical tool for evaluating dynamic balance during functional activities, was used. Findings demonstrated that the patients exhibited comparable levels of lower limb muscle strength and dynamic postural control, enabling them to maintain normal performance in physical activity tests (Chen et al., 2022; Lopes Ferreira et al., 2020).

#### 2.3.2 Assessment of pain characteristics

**FIGURE 2:**
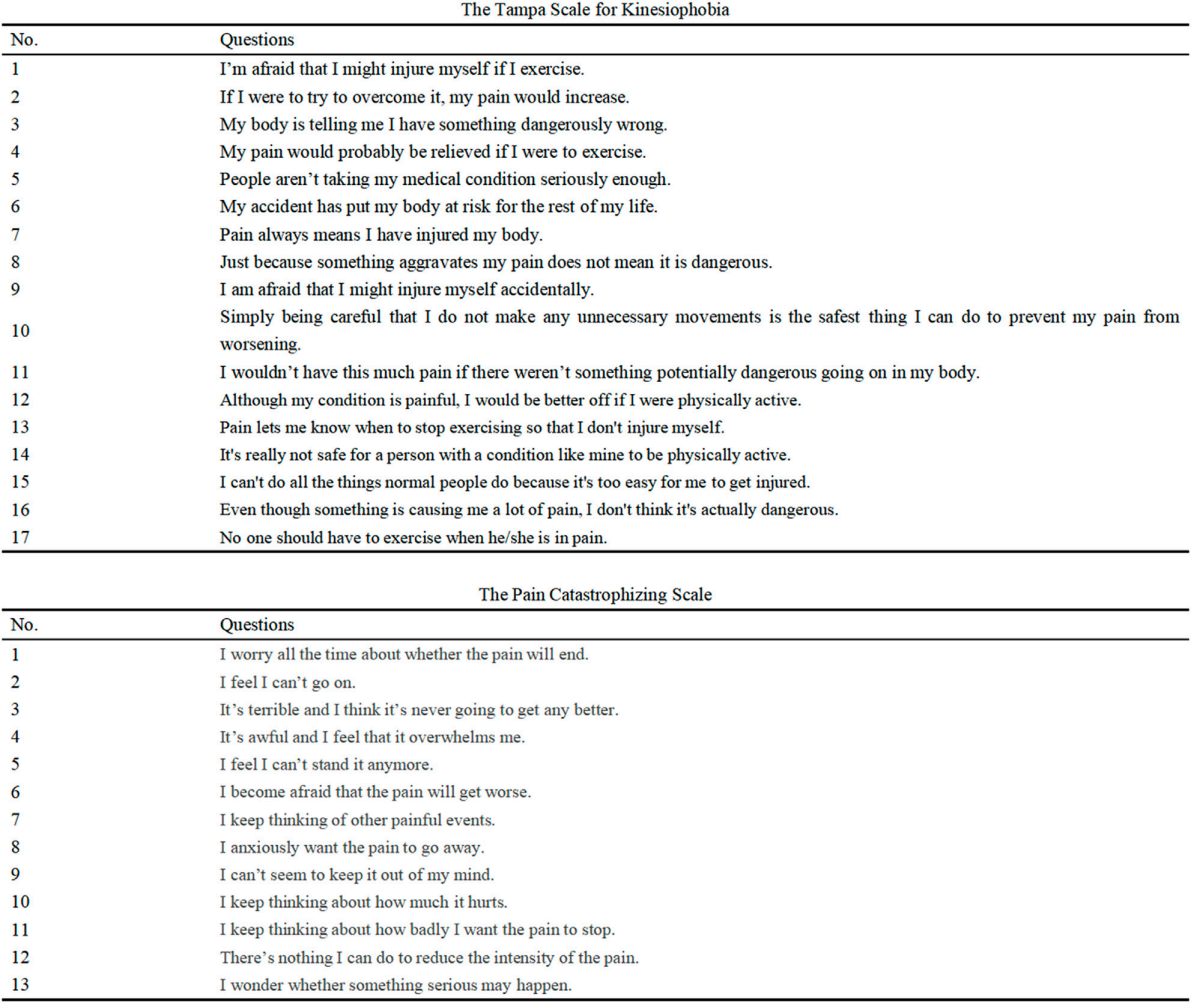
Pain scale image.

(1) VAS: Participants were asked to rate the intensity of knee joint pain experienced over the past 7 days on a 100-mm visual analog scale. The VAS was a horizontal line with endpoints labeled “no pain” and “worst pain imaginable”. Participants marked a point on the line to indicate their perceived pain intensity and the distance in millimeters from the “no pain” end was recorded (Tang et al., 2025). (2) Pain in knee flexion/extension (PKF/PKE) (Jonsson and Rasmussen-Barr, 2018): Active range of motion (ROM) was assessed to evaluate participant pain during knee flexion and extension (Luomajoki et al., 2007). Participants were seated on a treatment bed and asked to flex or extend their knee to its limit from a position of maximum flexion or extension, respectively. If the VAS score exceeded 30 mm during this movement, it was recorded as ‘yes’; otherwise, it was recorded as ‘no’. (3) Kinesiophobia: The Tampa Scale for Kinesiophobia (TSK) was used to assess participants’ fear-avoidance beliefs related to movement. Higher TSK scores indicate greater levels of kinesiophobia (Genç et al., 2025). (4) Pain catastrophizing: The Pain Catastrophizing Scale (PCS) was used to assess participants’ tendency to catastrophize pain. Higher PCS scores indicate greater levels of pain catastrophizing (Capelle et al., 2021). (5) Duration of pain (DP): A modified version of the Danish Standard Nordic Questionnaire was used to assess participants’ pain stages (Norheim et al., 2019). The questionnaire comprised two questions regarding pain duration and intensity. Pain duration was determined by the number of days participants experienced pain in the past 12 months. Participants with a VAS score exceeding 30 mm were further categorized using a 6-point scale: 1 for 0 days, 2 for 1–7 days, 3 for 8–30 days, 4 for 31–90 days, 5 for more than 90 days, and 6 for all remaining responses. Pain scale images are presented in Figure 2.

#### 2.3.3 Acquisition of motion patterns

A designated 8-m section of the field served as the test site. All participants were required to wear loose-fitting shorts and flat-soled trainers, utilizing a heel-to-toe gait transition throughout the test. To ensure optimal placement of the IMUs on the dorsal foot surface and prevent potential interference with toe-off maneuvers during the support phase, participants wore athletic shoes, with the sensors mounted on the top of the footwear (Yoshikawa et al., 2025). Given that self-selected walking speed is a reliable indicator of gait performance and is often employed to assess exercise capacity (Cabanas-Valdés et al., 2023), participants were instructed to maintain their habitual walking pace. Prior to the formal assessment, the tester provided a detailed explanation of the procedure and its requirements. To mitigate the influence of a learning effect, participants were encouraged to practice beforehand to establish consistent performance. All tests were conducted under the direct supervision of a researcher. The formal assessment consisted of three walks on level ground, each demanding at least three consecutive complete gait cycles. No injuries occurred during testing. However, 5 participants withdrew, reducing the final sample size to 104 out of the originally planned 109 subjects.

### 2.4 Selection of outcomes

#### 2.4.1 Temporal parameters

(1) Striding Time (ST): The duration between the moment a foot strikes the ground and the subsequent landing of the same heel. (2) Percentage of Swing Phase: The proportion of the swing phase within the entire gait cycle. (3) Step Frequency: The number of times the stride time is completed within 1 minute.

#### 2.4.2 Spatial parameters

(1) Joint Angles: The angles of the hip, knee, and ankle joints were measured during both the support and swing phases of walking. Joint angles were defined with respect to the position of the subject when standing calmly upright with their toes facing forward, which was designated as 0°. The real-time angle during movement was calculated as the angle relative to this calm upright position. (2) Step Length (SL): The distance between the right and left heels, normalized by leg length, which is defined as the distance from the anterior superior iliac spine to the medial malleolus.

#### 2.4.3 Derived parameters

(1) Coordination: The continuous relative phase method was employed to assess the coordination between adjacent lower limb links (thigh-calf, calf-foot) during support and swing phases. This technique facilitates comparisons of inter-segmental coordination by quantifying the phase relationship between neighboring joints at each time point during cyclic motion. To this end, the sagittal plane angles (θ) of the thigh, calf, and foot were normalized to a maximum value of ‘+1’, a minimum value of ‘-1’, and an intermediate value of ‘0’. Similarly, angular velocity (ω) was normalized to its maximum value. The calculation method is as follows:

$$\theta i=\left(\frac{2\left[\theta i-\min\theta i\right]}{\max\left(\theta i\right)-\min\left(\theta i\right)}\right)-1$$ (1)

$$\omega i=\left(\frac{\omega i}{\max\left\{\left|\omega i\right|\right\}}\right)$$ (2)

$$\varphi i=\arctan\left(\frac{\omega i}{\theta i}\right)$$ (3)

RPA=φ Proximal Link−φ Distal Link (4)

$$MARP=\frac{\sum_{i=1}^{P}\left|RPA\right|}{P}$$ (5)

The symbol φ represents the phase angle, while RPA denotes the relative phase angle. The Mean Absolute Relative Phase (MARP) indicates the average of the absolute values of the relative phase angles. The variable ‘i' corresponds to a specific point within the gait cycle. A smaller value of MARP signifies improved coordination between the links.

(2) Symmetry Index (SI): SI quantitatively measures the symmetry of a movement pattern and is calculated using the following formula:

$$SI=\frac{2\left(L-R\right)}{\left(L+R\right)}\times 100\%$$ (6)

Where, “L” and “R” denote the measurements of the left and right legs, respectively.

(3) Variability Index (VI): VI quantitatively measures the stability of a movement pattern and is calculated as follows:

$$VI=\frac{SD}{M}\times 100\%$$ (7)

Where, “SD” and “M” represent the standard deviation and the mean value of the relevant indicators, respectively.

(4) Shock Attenuation (SA): SA refers to the dynamics of the lower limb during movement. It denotes the reduction in both the magnitude and frequency of an impact as it travels from the distal part of the lower limb to the waist. SA is recognized as a significant factor influencing lower limb injuries and is calculated using acceleration parallel to the body’s longitudinal axis. The calculation is presented in the formula below:

$$SA=\left(1-\frac{PWA}{PTA}\right)\times 100\%$$ (8)

The Peak Waist Acceleration (PWA) refers to the maximum acceleration recorded by the waist IMU, while the Peak Tibia Acceleration (PTA) denotes the maximum acceleration recorded by the calf IMU. Higher values of these measurements indicate a greater capacity for shock attenuation.

### 2.5 Data analysis

Statistical Package for the Social Sciences (SPSS, v26) was employed to analyze the data. One-way ANOVA was utilized to compare the control, pain-free, and pain groups. The Shapiro-Wilk test indicated that the data were normally distributed. Post-hoc tests were conducted using the Least Significant Difference (LSD) method when the ANOVA chi-square criterion was met; otherwise, the Tamhane T2 test was employed. Data were presented as mean ± standard deviation (x̄ ± s). Due to the predominance of right-leg involvement in this cohort, the affected-side data were compared to right-leg data from a healthy walking population. To assess clinical significance for significant ANOVA results, *η* ^2^ was calculated with values ≥0.14 indicating large, 0.06 ≤ *η* ^2^ < 0.14 indicating medium, and 0.01 ≤ *η* ^2^ < 0.06 indicating small clinical significance (Cohen, 1992). The *η* ^2^ (eta squared) was calculated using SPSS for the results of the ANOVA, rather than for the *post hoc* tests.

Bivariate correlation analyses were performed to explore the relationship between pain characteristics and movement pattern parameters within the pain group. Point-biserial correlations were calculated between PKF and PKE and movement pattern indicators, assigning a value of 0 to no pain and 1 to pain. Spearman’s correlation coefficient was used to assess the relationship between pain characteristics (excluding PKF and PKE) and movement pattern indicators. Correlation coefficients were interpreted as follows (Akoglu, 2018): 0.00–0.09 = no correlation; 0.10–0.29 = weak correlation; 0.30–0.59 = moderate correlation; 0.60–0.79 = moderate strong correlation; and 0.80–1.00 = very strong correlation, with statistical significance set at *p* < 0.05.

## 3 Results

### 3.1 Differences in walking movement patterns between groups with and without pain

#### 3.1.1 Temporal parameters

Table 3 presents the results of the comparison of temporal parameters among the groups. A significant increase in stride time was observed in both the pain-free and pain groups compared to the control group (*p* < 0.05). Similarly, the pain group exhibited a significantly higher percentage of swing periods than the control group (*p* < 0.05). Conversely, a significant decrease in step frequency was found in both the pain-free and pain groups relative to the control group (*p* < 0.05). The aforementioned significant differences exhibited medium clinical significance (0.06 ≤ Cohen’s *η* ^2^ < 0.14).

**TABLE 3:** Comparison of temporal parameters among groups (x̄ ± s).

Indicators	Control group	Pain-free group	Pain group	ANOVA *p*	Post-hoc tests *p*	*η* ^2^
Striding time (s)	1.14 ± 0.04	1.24 ± 0.15	1.25 ± 0.17	0.002*	0.017*/0.000*/0.957	0.116
Percentage of swing period (%)	39.29 ± 2.46	38.92 ± 2.56	38.05 ± 1.97	0.047*	0.557/0.019*/0.128	0.060
Step frequency (step/min)	107.93 ± 9.55	96.83 ± 10.97	96.96 ± 16.62	0.002*	0.001*/0.001*/0.999	0.119

Post-hoc test *p*-values are ordered as follows: between the control and pain-free groups, between the control and pain groups, and between the pain-free and pain groups. * indicates statistical significance.

#### 3.1.2 Spatial parameters

Table 4 presents the results of the comparison of spatial parameters among groups. At the hip joint angle, the hip angle at heel strike (HAHS) and hip ROM were significantly greater in the control group than in the pain group (*p* < 0.05). Similar findings were observed for knee and ankle angles, with the knee angle at toe-off (KATO), knee ROM, ankle angle at heel strike (AAHS), and ankle ROM significantly greater in the control group than the pain-free and pain groups (*p* < 0.05). Additionally, KATO was significantly greater in the pain-free group than in the pain group (*p* < 0.05). Furthermore, SL was significantly greater in the control group than in the pain-free and pain groups (*p* < 0.05). The significant differences mentioned above exhibited medium to large clinical significance (Cohen’s *η* ^2^ ≥ 0.06).

**TABLE 4:** Comparison of spatial parameters among groups (x̄ ± s).

Indicators	Control group	Pain-free group	Pain group	ANOVA *p*	Post-hoc tests *p*	*η* ^2^
HAHS (°)	33.50 ± 4.88	32.50 ± 3.95	29.70 ± 7.56	0.022*	0.797/0.022*/0.117	0.073
HATO (°)	0.42 ± 5.90	−4.75 ± 8.12	−4.78 ± 11.95	0.053	0.059/0.053/0.990	
Hip ROM (°)	51.09 ± 10.94	46.61 ± 8.87	44.03 ± 10.75	0.016*	0.124/0.004*/0.327	0.079
KAHS (°)	14.93 ± 5.12	11.06 ± 8.47	11.56 ± 8.26	0.099	0.172/0.076/0.994	
KATO (°)	39.69 ± 13.13	32.88 ± 9.01	25.83 ± 11.68	0.000*	0.037*/0.000*/0.017*	0.214
Knee ROM (°)	69.64 ± 7.76	60.54 ± 9.63	62.42 ± 12.88	0.005*	0.002*/0.007*/0.866	0.101
AAHS (°)	5.33 ± 8.68	−0.66 ± 7.37	−3.01 ± 8.85	0.000*	0.012*/0.000*/0.274	0.154
AATO (°)	−11.44 ± 11.51	−6.93 ± 14.26	−7.57 ± 16.71	0.445	0.276/0.260/0.865	
Ankle ROM (°)	52.64 ± 17.58	38.51 ± 8.78	40.39 ± 11.04	0.000*	0.000*/0.000*/0.561	0.176
SL (%)	73.47 ± 2.82	67.73 ± 4.49	66.54 ± 4.34	0.000*	0.000*/0.000*/0.239	0.368

Post-hoc test *p*-values are ordered as follows: between the control and pain-free groups, between the control and pain groups, and between the pain-free and pain groups. HAHS, hip angle at heel strike; HATO, Hip angle at toe-off; ROM, range of motion; KAHS, knee angle at heel strike; KATO, Knee angle at toe-off; AAHS, ankle angle at heel strike; AATO, Ankle angle at toe-off. Post-hoc test p-values were ordered as follows: between the control and pain-free groups, between the control and pain groups, and between the pain-free and pain groups. * indicates statistical significance. Hip flexion is considered positive, while extension is regarded as negative. Similarly, knee flexion is positive, and knee extension is negative. Ankle dorsiflexion is positive, whereas plantarflexion is negative.

#### 3.1.3 Derived parameters

Table 5 presents the results of intergroup comparisons of the derived parameters. The SI (ST) and SI (SL) were significantly lower in the control group than in both the pain-free and pain groups (*p* < 0.05). Additionally, the VI (SL) was significantly lower in the control group than in the pain group (*p* < 0.05). Regarding coordination, thigh-calf MARP during the support phase was significantly greater in the control group than in the pain-free and pain groups (*p* < 0.05). Conversely, calf-foot MARP during the support phase was considerably smaller in the control group than in the pain group (*p* < 0.05). Additionally, calf-foot MARP during the swing phase was significantly smaller in the control group than in the pain-free group (*p* < 0.05). Furthermore, the SA was significantly greater in the control group than in both the pain-free and pain groups (*p* < 0.05). The significant differences mentioned above exhibited medium to large clinical significance (Cohen’s *η* ^2^ ≥ 0.06).

**TABLE 5:** Comparison of derived parameters among groups (x̄ ± s).

Indicators	Control group	Pain-free group	Pain group	ANOVA *p*	Post-hoc tests *p*	*η* ^2^
SI (ST, %)	2.24 ± 1.88	4.40 ± 2.58	3.84 ± 2.24	0.001*	0.001*/0.002*/0.321	0.126
SI (SL, %)	4.90 ± 2.29	7.66 ± 3.91	6.74 ± 3.64	0.010*	0.015*/0.020*/0.718	0.087
VI (ST, %)	4.55 ± 3.75	5.09 ± 1.98	5.07 ± 2.33	0.678	0.484/0.412/0.983	
VI (SL, %)	4.74 ± 1.53	5.69 ± 2.42	6.92 ± 2.97	0.001*	0.292/0.000*/0.183	0.127
Thigh-calf MARP during the support phase (°)	33.68 ± 3.55	27.35 ± 6.36	29.38 ± 6.94	0.001*	0.000*/0.002*/0.182	0.138
Calf-foot MARP during the support phase (°)	27.43 ± 4.80	31.46 ± 8.62	32.14 ± 8.22	0.024*	0.056/0.008*/0.718	0.072
Thigh-calf MARP during the swing phase (°)	36.53 ± 6.82	37.21 ± 6.56	37.42 ± 5.65	0.822	0.694/0.536/0.893	
Calf-foot MARP during the swing phase (°)	17.98 ± 6.14	23.46 ± 9.64	21.13 ± 7.46	0.035*	0.011*/0.077/0.228	0.064
SA (%)	37.45 ± 15.92	24.23 ± 13.60	24.73 ± 13.76	0.000*	0.001*/0.000*/0.889	0.145

Post-hoc test *p*-values are ordered as follows: between the control and pain-free groups, between the control and pain groups, and between the pain-free and pain groups. * indicates statistical significance.

The four MARP indicators are calculated from Equations 1 -5; the two SI indicators are calculated from Equation 6; the two VI indicators are calculated from Equation 7; and the SA indicator is calculated from Equation 8.

### 3.2 Correlations between pain characteristics and walking movement patterns in the pain group

#### 3.2.1 Temporal parameters

There were no significant associations between pain characteristics and temporal parameters (*p* > 0.05; Table 6).

**TABLE 6:** Correlation analysis between pain characteristics and temporal parameters.

Parameters	Striding time (s)		Percentage of swing period (%)		Step frequency (step/min)	
	*r*	*p*	*r*	*p*	*r*	*p*
PKF	−0.088	0.539	−0.120	0.400	−0.007	0.962
PKE	0.161	0.259	−0.106	0.457	−0.135	0.343
VAS (mm)	−0.081	0.570	0.138	0.334	0.008	0.953
TSK	0.130	0.362	−0.178	0.213	−0.149	0.298
PCS	−0.182	0.201	−0.022	0.881	0.188	0.186
DP	−0.047	0.743	0.266	0.059	−0.012	0.933

* indicates statistical significance.

#### 3.2.2 Spatial parameters

**FIGURE 3:**
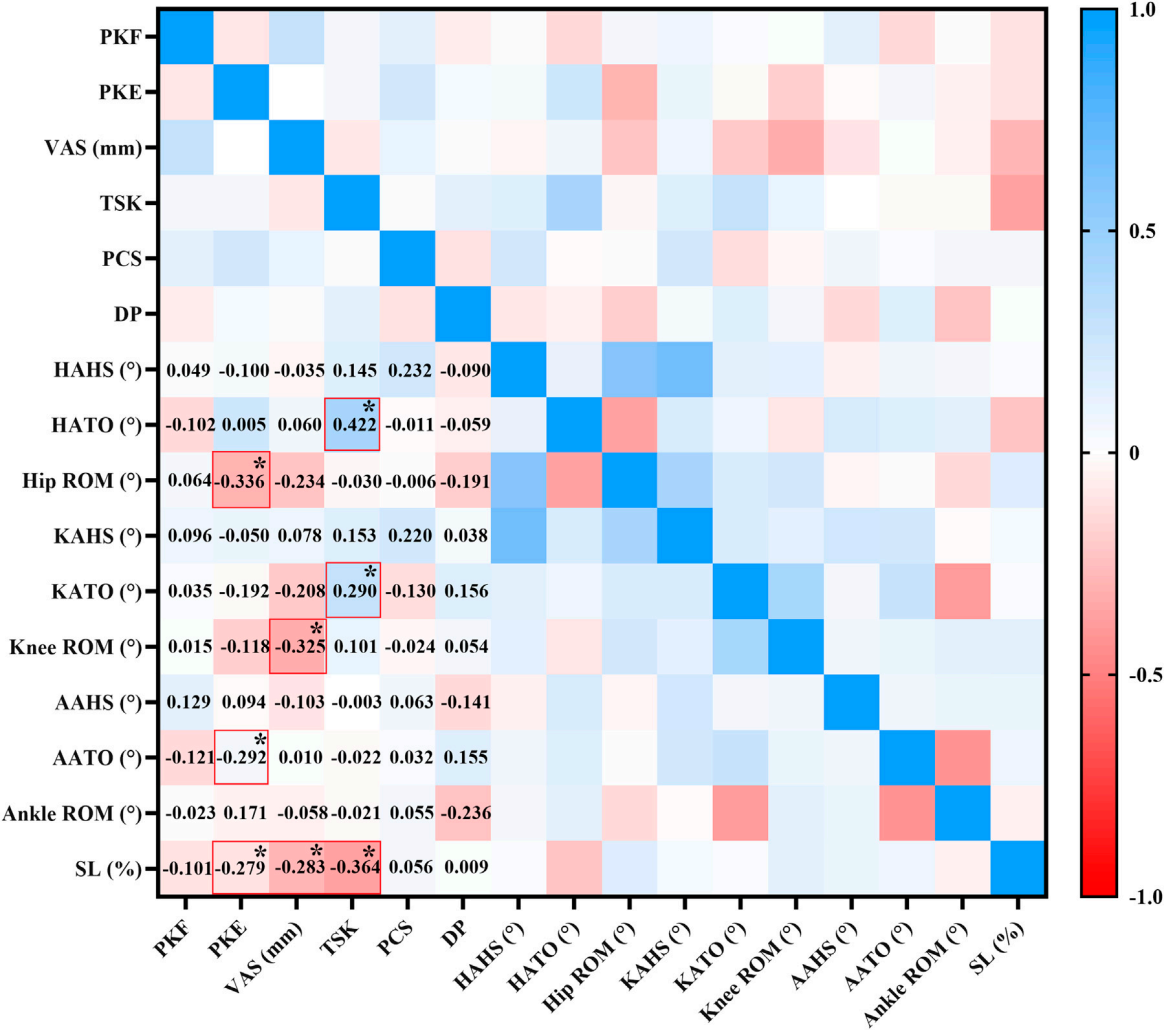
Correlation analysis between pain characteristics and spatial parameters. The numbers in the lower-left corner of the figure indicate the value of r. * indicates statistical significance.

Figure 3 illustrates the correlations between pain characteristics and spatial parameters. No significant correlations were found between pain characteristics and the HAHS, KAHS, AAHS or ankle ROM (*p* > 0.05). A moderate negative correlation was observed between the PKE and hip ROM (r = −0.336, *p* < 0.05), and weak negative correlations were observed between the PKE and AATO (r = −0.292, *p* < 0.05), and SL (r = −0.279, *p* < 0.05). Additionally, a moderate negative correlation was noted between the VAS and knee ROM (r = −0.325, *p* < 0.05), along with a weak negative correlation between VAS and SL (r = −0.283, *p* < 0.05). Furthermore, a moderate positive correlation was identified between the TSK and the HATO (r = 0.422, *p* < 0.05), a weak positive correlation with the KATO (r = 0.290, *p* < 0.05), and a moderate negative correlation with SL (r = −0.364, *p* < 0.05).

#### 3.2.3 Derived parameters

**FIGURE 4:**
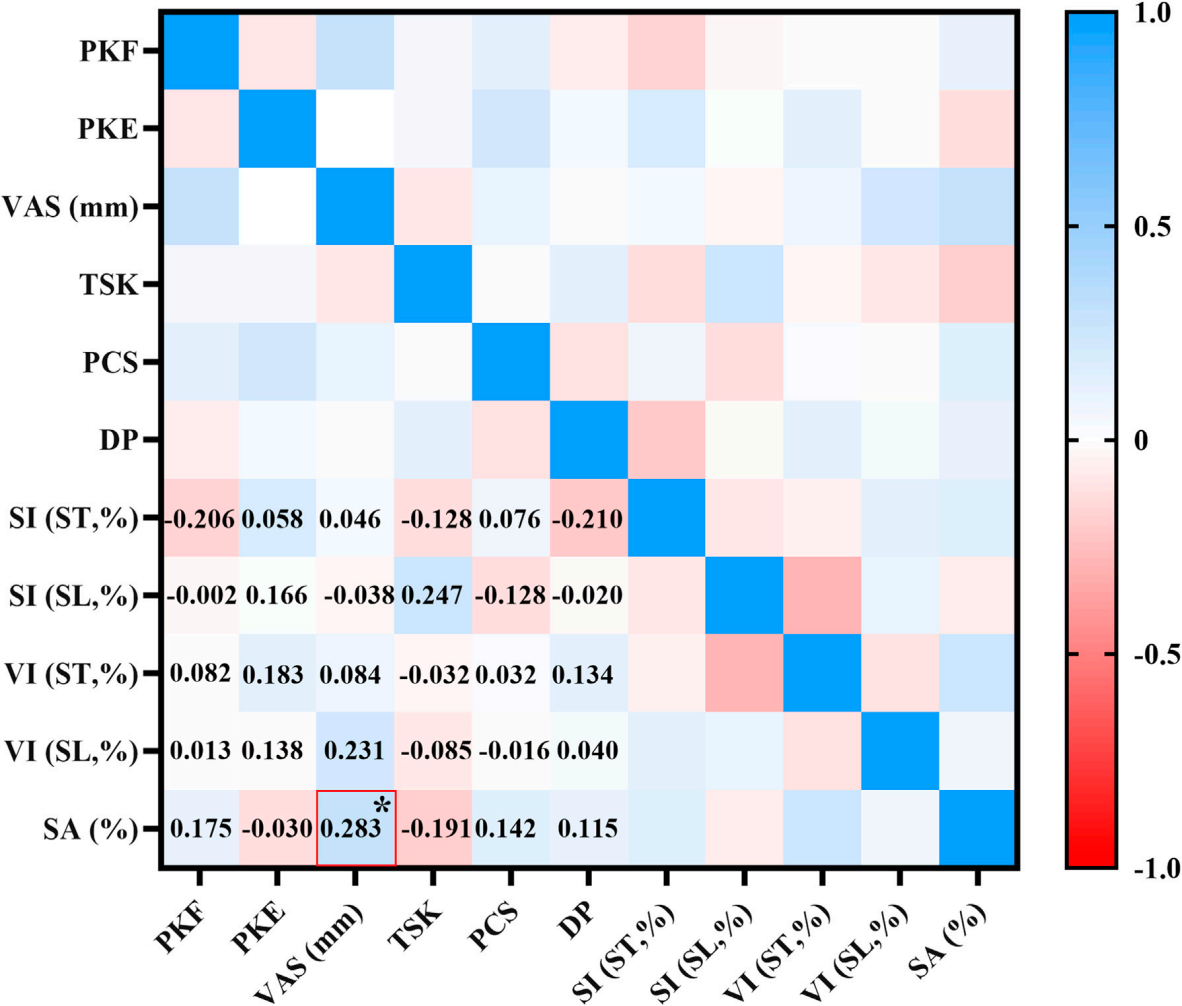
Correlation analysis between pain characteristics and derived parameters (SI/VI/SA). The numbers in the lower-left corner of the figure indicate the value of r. * indicates statistical significance.

Table 7 and Figure 4 present the correlations between pain characteristics and derived parameters. A moderate negative correlation was observed between PKE and thigh-calf MARP during the support phase (r = −0.303, *p* < 0.05), as well as a moderate positive correlation between TSK and calf-foot MARP during the swing phase (r = 0.356, *p* < 0.05), as detailed in Table 7. Figure 4 further indicates a weak positive correlation between VAS and SA (r = 0.283, *p* < 0.05). No significant correlations were found between the remaining parameters.

**TABLE 7:** Correlation analysis between pain characteristics and coordination.

Parameters	Thigh-calf MARP during the support phase (°)		Calf-foot MARP during the support phase (°)		Thigh-calf MARP during the swing phase (°)		Calf-foot MARP during the swing phase (°)	
	*r*	*p*	*r*	*p*	*r*	*p*	*r*	*p*
PKF	0.120	0.402	−0.001	0.994	0.182	0.201	0.141	0.325
PKE	−0.303	0.031*	0.100	0.487	−0.104	0.467	0.069	0.630
VAS (mm)	0.254	0.072	−0.188	0.186	0.018	0.903	0.101	0.480
TSK	−0.028	0.845	0.101	0.480	0.202	0.154	0.356	0.010*
PCS	0.054	0.704	−0.054	0.705	−0.068	0.633	−0.007	0.960
DP	0.138	0.335	−0.155	0.278	0.245	0.084	0.046	0.751

* indicates statistical significance.

## 4 Discussion

### 4.1 The impact of pain presence or absence on walking movement patterns

#### 4.1.1 Temporal-spatial parameters

Our findings suggest that pain, a subjective sensation, led to a shorter swing phase duration, reduced hip angle, and decreased knee angle of the affected limb during walking. During the swing phase, the lower limb joints generate a striding motion characterized by a greater amplitude of movement. However, patients with medial meniscus injuries exhibited a shortened swing phase of the affected leg due to significant pain, likely to avoid exacerbating joint discomfort associated with a greater range of motion. This observation was consistent with the finding that pain diminishes hip and knee mobility. Meniscal injuries often induce localized pain in the knee joint, directly impacting its mobility and indirectly affecting adjacent joints. Significant knee pain can reduce thigh swing and limit forward step range of motion, decreasing hip and knee angles (Mohamed and Alatawi, 2023). demonstrated that pain relief achieved through Kinesio taping combined with conventional physical therapy resulted in an increased knee flexion angle in patients with knee osteoarthritis, thereby supporting our findings. However, some studies, such as (Yang et al., 2022) ‘s research on patellofemoral joint pain syndrome, have not identified a significant relationship between knee pain and knee flexion angle during running and landing jumps, although pain did reduce knee abduction angle and patellofemoral joint loading, suggesting a protective mechanism. Discrepancies between our findings and previous studies may be attributed to differences in test operations and patient status. It's noteworthy that earlier studies did not utilize IMUs and focused on different types of diseases.

#### 4.1.2 Derived parameters

Our results indicated that variability and coordination during walking were influenced by pain, while the remaining derived parameters remained unaffected. Gait parameters are inherently dynamic, fluctuating over time. Indeed, the repeatability of walking can vary across trials, even under controlled environmental and external conditions. Variability in kinematic parameters beyond joint angles reflects inconsistency in lower limb movement and is a critical indicator of gait stability (Wang et al., 2024). Our tests revealed that significant pain contributes to decreased stability in patients’ walking patterns. Similar findings have been reported in other disorders (Mandeville et al., 2008). examined patients with knee osteoarthritis who underwent total knee arthroplasty while navigating obstacles both pre- and post-operatively (6 months post-surgery). Their results demonstrated that pain could adversely affect the stability of patients’ movements. Additionally (Lee et al., 2021), confirmed that gait instability in patients with herniated lumbar discs may be exacerbated by pain. These studies corroborate the findings presented in this paper.

MARP represents the coupling coordination between adjacent segments during walking. A smaller MARP value indicates greater cooperation between the lower limbs, which can help prevent excessive shifts in the center of gravity and loss of balance, thereby enhancing walking efficiency (Qu et al., 2024). In this study, pain caused changes in the coordination of the calf-foot in both the support and swing phases. It is generally accepted that the ankle primarily regulates movement strategy during the support phase, while the knee predominantly regulates it during the swing phase (Wang et al., 2021). Our findings demonstrated that pain influenced the ankle’s role in regulating coordination during both phases, indicating an alteration of the original motor strategies due to pain.

### 4.2 Relationship between pain characteristics and movement patterns in patients expe-riencing pain

Given the potential influence of heightened pain intensity and severe pain-related psychological factors on alterations in walking movement patterns, we conducted further correlation analyses.

#### 4.2.1 Temporal-spatial parameters

Our results indicated no correlation between pain characteristics and temporal parameters within the pain group. However, correlations with spatial parameters were observed. PKE was the pain characteristic most closely correlated with spatial parameters during walking. Throughout a complete gait cycle, the knee undergoes extension during both the support and swing phases. During the support phase, the knee extends while bearing weight. In the swing phase, it extends while the hip flexes forward and the foot lifts to find a suitable landing spot. Pain experienced during extension directly impacts knee extension. To mitigate knee pain during extension, patients may actively reduce the angle of knee extension. When this extension is insufficient, compensatory movements in neighboring joints may occur (Brunner and Rutz, 2013). Hip ROM reflects flexibility during walking, while ankle plantarflexion influences stability. When the toes are off the ground, the ankle joint is in plantarflexion. Increased plantarflexion can hinder the lifting of the lower limb at the beginning of the swing phase, causing the toes to remain too close to the ground and increasing the risk of falling (Sudo et al., 2021). In this study, significant pain during knee extension was associated with decreased hip ROM and increased ankle plantarflexion angle, negatively impacting flexibility and balance during walking. In addition, knee extension accompanied by pain reduced SL during walking, which is linked to decreased hip ROM. Sufficient hip ROM is crucial for achieving an appropriate SL while walking. A direct consequence of limited hip ROM is a shorter SL, decreased walking efficiency, and increased energy expenditure.

VAS was negatively correlated with knee ROM and SL during walking in patients with medial meniscus injuries. This alteration may represent a strategy employed by patients to avoid inducing high-intensity pain, which is more likely to occur with a greater ROM. However, this adjustment is not conducive to optimal gait development. Current research on the effect of pain intensity on movement patterns is inconsistent. For instance (Sarridou et al., 2024), found that pain intensity was negatively correlated with knee mobility in patients following total knee replacement. (Subbarayalu and Ameer, 2017), found that smaller cranial vertebral bone angles corresponded to greater neck pain intensity in patients with postural neck pain. Conversely (Schmidt et al., 2019), found different patterns - knee pain increased with greater external rotation in patellofemoral syndrome, while hip pain worsened with more adduction and internal rotation in chronic hip pain patients. The discrepancies in findings may be attributed to the different types of diseases and movement tasks. In the former case, pain limited joint motion, whereas in the latter, it resulted in excessive movement in the coronal and horizontal planes, thereby reducing stability. The impact of pain on movement patterns is not uniform, making it impractical to generalize findings to all patients experiencing pain. It is essential to differentiate between types of diseases and locations of pain, as well as to establish a rigorous research protocol to enhance the quality of the study.

Kinesiophobia is a significant emotional response characterized by an intense fear of re-injury during physical activity, often leading many patients to avoid sports altogether (Wlazlo et al., 2025). The present study revealed that higher scores on the TSK were associated with decreased hip extension when patients’ toes were off the ground, negatively impacting their ability to achieve an adequate SL. Although there is a lack of literature specifically addressing the effects of kinesiophobia on movement patterns in patients with meniscus injuries, studies have shown that in individuals with anterior cruciate ligament (ACL) injuries, kinesiophobia correlates with their biomechanical performance during movement (Larsen et al., 2024). Patients with ACL injuries who exhibit higher kinesiophobia scores often demonstrate weaker quadriceps strength (Lentz et al., 2014; Paterno et al., 2018) and reduced flexion angles in the knee, hip, and trunk during exercise (Trigsted et al., 2018). It has been hypothesized that these altered movement patterns are driven more by a fear of pain than by the actual intensity of pain or the level of disability experienced, which aligns with the results of this study. This result suggests that pain education addressing kinesiophobia is essential. A 4-week pain neuroscience education has been shown to be effective in patients with shoulder pain (Delgado-Gil et al., 2025) and can be generalized to patients experiencing knee pain.

#### 4.2.2 Derived parameters

Next, we found that the pain characteristics associated with the derived parameters included the VAS, PKE, and TSK. A higher VAS score correlated with greater SA. During exercise, the body employs various SA strategies to manage the impact from the ground. These strategies can be categorized into passive and active mechanisms. Passive mechanisms include fat pads, skin, bones, ligaments, and tendons, while active mechanisms involve centrifugal muscle contractions, among others. Together, these mechanisms work to mitigate the impact force from the ground. The results from the previous section of this study indicated that SA in patients with medial meniscus injuries was significantly lower than that of healthy subjects. This difference is related to the reduced knee flexion angle caused by meniscus injuries (Edwards et al., 2012), which is a direct consequence of the injuries. In contrast, the results in this section demonstrated that an elevated VAS score led to increased walking stability, allowing the lower extremities to absorb more ground impact—a favorable outcome in the context of pain management.

The results indicated an association between PKE and MARP in the thigh-calf complex during the support phase. Previous findings demonstrated that knee extension pain during walking is linked to a reduction in joint angle, potentially serving as a protective mechanism to mitigate joint loading and alleviate pain. Interestingly, while this decrease in joint mobility may compromise movement efficiency, it enhances the coupling between adjacent lower limb segments and improves thigh-calf coordination during the stance phase.

The present study also revealed an association between kinesiophobia and calf-foot MARP during the swing phase. Prior research has demonstrated that individuals with elevated TSK levels engage in fewer physical activities during their daily lives (Wlazlo et al., 2025). Prolonged periods of inactivity can impair the ability to perform appropriate movements, and a lack of motor experience ultimately leads to reduced coordination. There is currently no literature examining the relationship between kinesiophobia scores and walking coordination. However, similar studies have confirmed that kinesiophobia is associated with decreased movement quality (Luque-Suarez et al., 2019; Mekonnen et al., 2025; Uçmak and Kilinç, 2024), consistent with the present study’s findings. Nonetheless, none of the studies in this literature focused on meniscal injuries, leading to insufficient support for the evidence.

We found that the presence of pain, a subjective sensation, led to alterations in walking movement patterns among patients with medial meniscus injuries. A shorter swing phase proportion and reduced joint angles characterized these alterations. These changes in movement patterns were not simply a decline in quality; instead, they represented an adaptive response aimed at protecting the individual in the presence of pain. While these adaptive changes had unfavorable aspects, such as increased variability in walking, they also had favorable aspects, including enhanced coordination and shock attenuation during ambulation. By analyzing the relationship between pain characteristics and movement patterns, we can infer that internal factors contributing to pain-induced changes in walking patterns may include higher pain intensity and more severe pain-related psychological factors. Therefore, in the diagnosis and treatment of patients with medial meniscus injuries, attention should be paid to these pain characteristics for personalized diagnosis and treatment. Pain neuroscience education should be employed to address kinesiophobia. We recommend the introduction of IMUs as biofeedback devices for retraining movement patterns during rehabilitation. Additionally, the angles of the hip, knee, and ankle during the swing phase should be a primary focus in retraining.

Future research could encompass two directions: pain management and movement pattern retraining. One direction would be to validate the role of pain relief through pain medication or pain neuroscience education, and the other would be to conduct movement pattern retraining to validate its effectiveness. Multidisciplinary interventions should be considered when conducting randomized controlled trials to help patients face their irrational fear of movement. It is essential to take into account each patient’s unique biological, psychological, and social experiences related to pain and kinesiophobia (Bordeleau et al., 2022).

### 4.3 Limitation

Our study did not stratify subjects by meniscal injury severity and injury pattern, limiting its ability to address this variable. Future studies should concentrate on this section to further refine the classification and typology of meniscal injuries, particularly in relation to pain levels.

## 5 Conclusion

Medial meniscus injury-induced pain has several adverse effects, including prolonged walking swing periods, reduced angulation, and increased variability while positively influencing coordination and shock attenuation. Pain characteristics, such as pain intensity, kinesiophobia, and pain in knee extension, contribute to these changes. These changes in movement patterns should be taken seriously in clinical practice. Therapists should focus on alleviating the patient’s pain symptoms and conduct gait retraining. The angles of the hip, knee, and ankle during the swing phase should be a primary focus in retraining. Future research should investigate the impact of pain interventions on movement patterns to inform the development of rehabilitation in clinical settings.

## Data Availability

The original contributions presented in the study are included in the article/supplementary material, further inquiries can be directed to the corresponding authors.
